# Escenario futuro de la diabetes mellitus tipo 2 estimado con un modelo de simulación dinámico predictivo

**DOI:** 10.26633/RPSP.2017.93

**Published:** 2017-09-29

**Authors:** Darío Gaytán-Hernández, Sandra Olimpia Gutiérrez-Enríquez, Aracely Díaz-Oviedo, Claudia Elena González-Acevedo, Magdalena Miranda-Herrera, Luis Eduardo Hernández-Ibarra

**Affiliations:** 1 Facultad Enfermería Universidad Autónoma de San Luis Potosí-Posgrado San Luis Potosí Mexico Facultad Enfermería, Universidad Autónoma de San Luis Potosí-Posgrado, San Luis Potosí, San Luis Potosí, Mexico.

**Keywords:** Diabetes mellitus, factores de riesgo, predicción, México, Diabetes mellitus, risk factors, forecasting, Mexico, Diabetes mellitus, fatores de risco, previsões, México

## Abstract

**Objetivo.:**

Desarrollar un modelo dinámico predictivo para estimar escenarios futuros de la tasa de incidencia de diabetes mellitus tipo 2 (TIDM2).

**Métodos.:**

Se realizó un estudio ecológico retrospectivo durante el periodo 2013-2015 en la ciudad de San Luis Potosí, México. Se analizaron datos oficiales secundarios de los 58 municipios que integran el estado de San Luis Potosí. Se aplicó la correlación lineal, la regresión lineal múltiple, ecuaciones estructurales, y se desarrollaron cuatro submodelos dinámicos predictivos: TIDM2, población urbana, viviendas particulares habitadas que cuentan con televisión y población de 45-49 años de edad. Se desarrolló también un modelo holístico.

**Resultados.:**

El modelo estructural explica 27,2% del total de la varianza de la diabetes mellitus tipo 2. El porcentaje de viviendas habitadas que cuentan con televisión pesan 4,46 unidades no estándar sobre la diabetes, el de población urbana, 2,84 y el de población de 45-49 años, 156,69. Los escenarios estimados de la TIDM2 por 100 000 habitantes, para los años 2015, 2020, 2025 y 2030 fueron 1 052,4, 1 413,7, 1 850,1 y 2 351,1 respectivamente.

**Conclusión.:**

El escenario de la TIDM2 muestra un crecimiento exponencial del año 2000 al 2030. Los factores de riesgo según el peso que representan para la ocurrencia de la enfermedad fueron: población de 45-49 años, viviendas particulares habitadas que cuentan con televisión y población urbana.

## Abstract (en)

**Objective.:**

Develop a predictive dynamic model to estimate future scenarios for the incidence rate of type 2 diabetes mellitus (T2DM).

**Methods.:**

A retrospective ecological study was conducted in 2013-2015 in the city of San Luis Potosí, Mexico. Secondary official data from the 58 municipalities making up the state of San Luis Potosí were analyzed. Linear correlation, multiple linear regression, and structural equations were carried out, and four predictive dynamic submodels were developed: T2DM, urban population, inhabited private dwellings that have television, and population aged 45-49 years. A holistic model was also developed.

**Results.:**

The structural model explains 27.2% of total variance in type 2 diabetes mellitus. Percentage of inhabited dwellings that have television weighs 4.46 non-standard units on diabetes; that of urban population, 2.84; and that of population aged 45-49 years, 156.69. Estimated scenarios for T2DM per 100 000 population for the years 2015, 2020, 2025, and 2030 were 1,052.4, 1,413.7, 1,850.1, and 2,351.1 respectively.

**Conclusion.:**

The T2DM scenario shows exponential growth from 2000 to 2030. Risk factors according to the weight they represent in occurrence of the disease were: population aged 45-49 years, inhabited private dwellings that have television, and urban population.

## RESUMO

**Objetivo.:**

Desenvolver um modelo dinâmico preditivo para estimar cenários futuros da taxa de incidência de diabetes mellitus tipo 2.

**Métodos.:**

Foi realizado um estudo ecológico retrospectivo no período de 2013 a 2015 na cidade de San Luis Potosí, México. Foram analisados dados oficiais secundários dos 58 municípios que fazem parte do Estado de San Luis Potosí. Foi feita a análise de correlação linear, regressão linear múltipla e equações estruturais e construídos quatro submodelos dinâmicos preditivos: diabetes mellitus tipo 2, população urbana, domicílios particulares permanentes com televisão e população com idade de 45–49 anos. Foi também desenvolvido um modelo holístico.

**Resultados.:**

O modelo estrutural explica 27,2% do total da variança da diabetes mellitus tipo 2. A porcentagem de domicílios permanentes com televisão tem o peso de 4,46 unidades não padronizadas na diabetes, 2,84 na população urbana e 156,69 na população de 45–49 anos. Os cenários estimados da diabetes mellitus tipo 2 por 100.000 habitantes para 2015, 2020, 2025 e 2030 foram de 1.052,4, 1.413,7, 1.850,1 e 2.351,1 respectivamente.

**Conclusão.:**

O cenário da diabetes mellitus tipo 2 mostra um crescimento exponencial de 2000 a 2030. Os fatores de risco segundo o peso representado na ocorrência da doença foram população com 45–49 anos, domicílios particulares permanentes com televisão e população urbana.

La prevalencia de diabetes ha tenido un crecimiento sostenido y alarmante en el mundo. En 2014 había 387 millones de personas con diabetes en el mundo y se espera que en 2035 el número aumentará hasta 592 millones (1). En México, el incremento de la prevalencia también ha sido constante: la proporción de adultos con diagnóstico médico previo de diabetes fue de 5,8% en 2000, 7% en 2006 y 9,2% en el 2012 (2), y en el estado de San Luis Potosí, la prevalencia de diabetes mellitus en adultos de 20 y más años de edad pasó de 6,2% en 2006 a 10% en 2012 (3).

Existen múltiples factores que se han asociado con un aumento del riesgo de diabetes, como el sobrepeso, la obesidad, la edad, el sexo y, entre los genéticos, pertenecer a una raza determinada. Además, factores de tipo ambiental y conductuales tales como el nivel educativo, el ingreso, la urbanización, el acceso a servicios de salud y los estilos de vida también se han asociado con el aumento de dicho riesgo (4, 5).

Se han realizado diferentes estudios para analizar los factores asociados con la diabetes tipo 2, como uno en que se observó que el porcentaje de diabéticos aumentó sistemáticamente con la edad: de 1,69% en diabéticos entre 21 y 30 años a 20,9% en el de 61 y más años (6). En otras investigaciones se comprobó que en los residentes en áreas urbanas la prevalencia de diabetes era más alta que en los que vivían en zonas rurales (7, 8). También se ha observado que dedicar dos horas por semana o más a ver la televisión puede aumentar el riesgo de diabetes: 1,23 veces cuando se ve cinco horas y 2,0 veces si se ve 40 horas por semana (9).

Los estudios citados ofrecen pruebas científicas sobre la relación de algunos factores con el riesgo de desarrollar diabetes mellitus, sin considerar la evolución en el tiempo y en el espacio tanto de los factores de riesgo como de la diabetes, es decir, los análisis se han realizado desde una perspectiva bivariada sin tener en cuenta las posibles relaciones conjuntas y simultáneas entre ellos. Además, la presencia simultánea de varios factores de riesgo tiene un efecto no sólo aditivo, sino multiplicativo en el aumento del riesgo respecto a cada factor por separado (10).

El objetivo del presente trabajo es estimar y analizar escenarios futuros probables de la tasa de incidencia anual de la diabetes mellitus tipo 2 (TIDMT2) en el estado de San Luis Potosí, mediante el desarrollo de un modelo dinámico predictivo con enfoque holístico, considerando las relaciones multivariadas de algunos de los principales factores que la determinan, el peso que cada uno representa para la enfermedad estudiada, así como la evolución en el tiempo y en el espacio de dichos factores.

## MATERIALES Y MÉTODOS

Se realizó un estudio ecológico retrospectivo durante el periodo 2013-2015 en la ciudad de San Luis Potosí, México. Se analizaron bases de datos secundarias con indicadores oficiales de los 58 municipios que integran el estado de San Luis Potosí, correspondientes a los años 2000, 2005 y 2010, y 174 registros que conformaron el total de los datos existentes en los años anteriormente mencionados.

Se estimaron y analizaron escenarios futuros de la TIDMT2 por 100 000 habitantes en la población de 20 y más años de edad y se tomó como base la evidencia científica de los factores causales y su evolución en el tiempo. Se trabajó con los siguientes indicadores disponibles.

En la población de 20 y más años de edad: TIDMT2 (11), porcentaje de población femenina (PF) (12), porcentaje de población masculina (PM) (12), porcentaje de población urbana (vive en localidades ≥ 2 500 habitantes) (PU) (12), porcentaje de población con educación secundaria incompleta (PESI) (12), porcentaje de población sin derechohabiencia a servicios de salud (PSDSS) (12), y porcentaje de población que habla alguna lengua indígena (PHALI) (12).

En la población general: número de habitantes por automóvil registrado en circulación (NHPARC) (13), porcentaje de población ocupada que gana hasta 2 salarios mínimos (POGH2SM) (14, 15), índice de marginación (IM) (14, 15), e índice de rezago social (IRS) (16). Otros indicadores fueron el porcentaje de población de 20 a 44 años de edad (P20-44AE) (12), el porcentaje de población de 45 a 49 años de edad (P45-49AE) (12), el de población de 50 a 59 años de edad (P50-59AE) (12), el de población de 60 a 64 años de edad (P60-64AE) (12), el de de población de 65 y más años de edad (P65YMAE) (12), y el de viviendas particulares habitadas que cuentan con televisión (VPHTV) (12).

Se calcularon los coeficientes de correlación lineal de Person entre la TIDMT2 y cada uno de los indicadores. En los análisis posteriores se eliminaron los indicadores con correlaciones no significativas y el indicador población masculina por presentar una correlación perfecta con el indicador población femenina. Con ello quedaron 10 indicadores.

Posteriormente, se construyó un modelo de regresión lineal múltiple para identificar los factores asociados con la TIDMT2, así como la carga que representan. La independencia entre las variables independientes se validó mediante el estadístico de Durbin Watson (valores ≈ 2 indican independencia entre los residuos) (17). La normalidad de las variables se comprobó con la media de los residuos (media = 0) y la no colinearidad, mediante la tolerancia y los factores de inflación de la varianza (tolerancia < 0,01 y factor de inflación de varianza > 10 indican multicolinearidad) (18). Para realizar estos cálculos se utilizó el programa PASW Statistics versión 18.

El modelo se construyó con los indicadores TIDMT2 como variable dependiente y población de 45-49 años de edad, población urbana y viviendas particulares habitadas que cuentan con televisión como variables independientes, dado que fueron las únicas asociadas significativamente con la TIDMT2. Para validarlo, se desarrolló un modelo estructural confirmatorio con la técnica multivariante de los modelos de ecuaciones estructurales con el programa AMOS versión 20, estimado con el procedimiento máxima verosimilitud, en el cual se consideraron las múltiples relaciones entre los factores de riesgo y el error de medición de la variable dependiente.

Para facilitar en el modelo dinámico predictivo la interpretación del peso que cada indicador representa para la TIDMT2 se utilizó la solución no estandarizada. Del modelo de ecuaciones estructurales se obtuvo el porcentaje de la varianza de la TIDMT2 que es explicado (*porcentaje explicado*) por los factores de riesgo y la carga que cada factor tiene sobre dicha tasa (*carga factor*).

Posteriormente, se construyó un modelo dinámico predictivo para estimar escenarios futuros de la TIDM2. Para ello, se desarrollaron cuatro submodelos dinámicos predictivos con el software Vensim, utilizando los datos disponibles (valores observados) a nivel estatal y la carga que cada factor representa para esta tasa identificados por el modelo de ecuaciones estructurales. Las simulaciones se realizaron a partir de 2000 y hasta 2030. Las simulaciones con el submodelo TIDM2 se realizaron con datos procedentes de los otros tres submodelos (población urbana, viviendas particulares habitadas que cuentan con televisión y población de 45-49 años de edad).

Para estimar las constantes de las tasas de cambio de los grupos de edad 0-19, (CTC0-19), 20-44 (CTC20-44), 45-49 (CTC45-49) y de 50 y más años de edad (CTC50+), se utilizaron proyecciones del periodo 1990-2030 realizadas por el CONAPO (19) y las siguientes fórmulas:

CTC0-19 = Población de 19 años/Población de 0-19 años
CTC20-44 = Población de 44 años/Población de 20-44 años
CTC45-49 = Población de 49 años/Población de 45-49 años
CTC50+ = Población de 50 años/Población de 50 y más años

Para poner una sola constante de la tasa de cambio en cada grupo de edad en el modelo, se calculó el promedio de las constantes de las tasas de cambio en los años considerados con datos observados.

Las constantes de las tasas de cambio de la mortalidad en la población de 50 y más años (CTCM50+), de la población urbana (CTCPU), de las viviendas particulares habitadas totales (CTCVPHT) y de las viviendas particulares habitadas que cuentan con televisión (CTCVPHTV) se estimaron con la fórmula:

$$\text{ConstanTe }=({\left(\text{ Valorf }/\text{ Valori }\right)}^{\wedge }(1/\text{ periodo de años) }-1$$

donde, Valorf es el valor numérico que corresponde al indicador analizado en el año final del periodo considerado, *Valori*, el valor numérico que corresponde al indicador analizado en el año inicial del mismo periodo, y *periodo de años*, el número de años comprendidos en el periodo considerado (20).

Para construir las tablas de las tasas de emigración se utilizaron proyecciones del periodo 1990-2030 (19). Los valores iniciales de cada factor son los observados en el año 2000.

Las tasas de cambio de la población de los grupos de edad 0-19, 20-44, 45-49 y 50 y más años se denotan como (TC0-19), (TC20-44), (TC45-49) y (TC50+), respectivamente; las de la mortalidad de la población de 50 y más años, como (TCM50+), las de las viviendas particulares habitas totales, como (TCVPHT), las de las viviendas particulares habitadas que cuentan con televisión, como (TCVPHTV), y la tasa de cambio de la población urbana, como (TCPU). Asimismo, las viviendas particulares habitadas totales se denotan como (VPHT).

La función Time está definida por el software Vensim para cambiar o ajustar el periodo de tiempo de la simulación. La variable Regulador es auxiliar, para mantener el control de la simulación, y los comandos IF THEN ELSE son estructuras de control del programa Vensim. Las fórmulas y las funciones utilizadas en cada submodelo se incluyen en el cuadro 1. Los factores se estimaron con las siguientes fórmulas:

P45-49AE = Población de 45-49/Población 20 y más años
PU = Población urbana/Población 20 y más años
VPHTV = Viviendas particulares habitadas que cuentan con TV/Viviendas particulares habitadas totales

La estimación de los efectos individuales de cada factor sobre la TIDM2 se expresa en las fórmulas:

Efecto población 45-49 = (P45-49AE-Valori)*Carga factor
Efecto viviendas con TV = (VPHTV-Valori)*Carga factor
Efecto población urbana = (PU-Valori)* Carga factor

La suma de los efectos se estimó con la siguiente fórmula:

Suma de efectos = ((Efecto población 45-49 + Efecto población urbana + Efecto viviendas con TV)*100)/Porcentaje explicado

Las pendientes se calcularon con los valores estimados por la simulación con la función PENDIENTE de Excel.

**CUADRO 1.** Fórmulas y funciones utilizadas para estimar los submodelos

Submodelo	Fórmula
**Población de 45-49 años**	TC0-19 = CTC0-19*Población de 0-19
	Tasa emigración 0-19 = Tabla tasa emigración 0-19(Time)
	Emigración 0-19 = Tasa emigración 0-19*Población de 0-19
	Población de 0-19 = TC0-19 – TC20-44 - Emigración 0-19
	TC20-44 = Población de 0-19*CTC20-44
	Tasa emigración 20-44 = Tabla tasa emigración 20-44(Time)
	Emigración 20-4 =Tasa emigración 20-44*Población de 20-44
	Población de 20-44 = TC20-44 – TC45-49 - Emigración 20-44
	TC45-49 = Población de 20-44*CTC45-49
	Tasa emigración 45-49 = Tabla tasa emigración 45-49(Time)
	Emigración 45-49 = Tasa emigración 45-49*Población de 45-49
	Población de 45-49 = TC45-49-(TC50+)-Emigración 45-49
	TC50+ = Población de 45-49*CTC50+
	Tasa emigración 50 y más = Tabla tasa emigración 50 y más (Time)
	Emigración 50 y más = Tasa emigración 50 y más*Población de 50 y más
	Población de 50 y más = (TC50+)-(TCM50+)-Emigración 50 y más
	TCM50+ = (CTCM50+)*Población de 50 y más
	Población 20 y más = Población de 20-44 + Población de 45-49 +Población de 50 y más
**Población urbana**	Regulador urbana = IF THEN ELSE(Población urbana>=Población 20 y más,0,1)
	TCPU = CTCPU*Población urbana*Regulador urbana
	Población urbana = TCPU
**Viviendas particulares habitadas que cuentan con televisión**	TCVPHT = CTCVPHT*Viviendas particulares habitadas totales
	Viviendas particulares habitadas totales = TCVPHT
	Regulador VPHTV = IF THEN ELSE(Viviendas particulares habitadas que cuentan con televisión ≥ Viviendas particulares habitadas totales,0,1)
	TCVPHTV = CTCVPHTV*Regulador VPHTV* Viviendas particulares habitadas que cuentan con TV
	Viviendas particulares habitadas que cuentan con TV = TCVPHTV
**Tasa de incidencia de diabetes mellitus tipo 2**	Tasa de incidencia de diabetes mellitus tipo 2 = Suma de efectos

***Fuente:*** elaboración propia.

***Nota:*** las constantes de las tasas de cambio son: CTC0-19: de la población de 0 a 19 años de edad; CTC20-44: de la población de 20 a 44 años de edad; CTC45-49: de la población de 45 a 49 años de edad; CTC50+: de la población de 50 y más años; CTCM50+: de la mortalidad en población de 50 y más años de edad; CTCPU: de la población urbana de 20 y más años; CTCVPHT: de las viviendas particulares habitadas totales; CTCVPHTV: de las viviendas particulares habitadas que cuentan con televisión; Tasas de cambio TC0-19: de la población de 0 a 19 años de edad; TC20-44: de la población de 20 a 44 años de edad; TC45-49: de la población de 45 a 49 años de edad; TC50+: de la población de 50 y más años; TCM50+: de la mortalidad en población de 50 y más años; TCPU: de la población urbana de 20 y más años; TCVPHT: de las viviendas particulares habitadas totales, y TCVPHTV: de las viviendas particulares habitadas que cuentan con televisión.

Los indicadores población urbana y población de 45-49 años se validaron con los valores observados y los estimados por la simulación mediante el coeficiente de correlación de Pearson. Para los indicadores viviendas particulares habitadas que cuentan con televisión y TIDM2 se realizó una validación visual con el gráfico de dispersión. Se consideraron como valores observados las proyecciones propias para los años 2001, 2002, 2003, 2004, 2006, 2007, 2008 y 2009 en los indicadores población de 45-49 años y población urbana. Para el indicador viviendas particulares habitadas que cuentan con televisión se consideraron datos de 2000, 2005 y 2010, y para la TIDM2 se consideraron valores observados en todo el periodo 2000-2010.

Para el procesamiento y el análisis de los datos se utilizó el programa estadístico PASW Statistics versión 18.

## RESULTADOS

La estimación obtenida con la regresión lineal múltiple mostró que sólo tres indicadores contribuyen significativamente a explicar la varianza de la TIDM2, como se describe en la siguiente ecuación:

TIDM2 = 156,69 (población de 45-49) + 2,84 (población urbana) + 4,6 (viviendas particulares habitadas que cuentan con televisión) – 1148,31

Respecto al cumplimiento de los supuestos, la tolerancia de cada variable fue > 0,01 y su correspondiente factor de inflación de la varianza, < 10, con lo cual se valida la no colinearidad, mientras que la independencia entre las variables independientes se verificó con el estadístico Durbin Watson (DW = 1,942). Asimismo, la media de los residuos fue igual a 0, con lo cual la normalidad queda comprobada.

**FIGURA 1.**
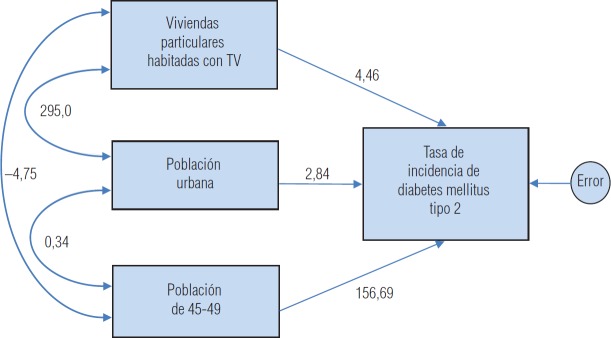
Modelo estructural confirmatorio de la tasa de incidencia de la diabetes mellitus tipo 2

El análisis confirmatorio con ecuaciones estructurales indica que el modelo explica 27,2% del total de la varianza de la TIDM2 (figura 1). Por otro lado, el modelo confirmatorio corrobora el resultado de la regresión lineal múltiple: las cargas de cada factor sobre la TIDM2 fueron estadísticamente significativas (viviendas particulares habitadas que cuentan con televisión p = 0,008, población urbana p < 0,001, y población de 45-49 p = 0,003). Las covariancias también fueron significativas (p < 0,001) excepto entre la población urbana y población de 45-49 (*p* = 0,847).

**FIGURA 2.**
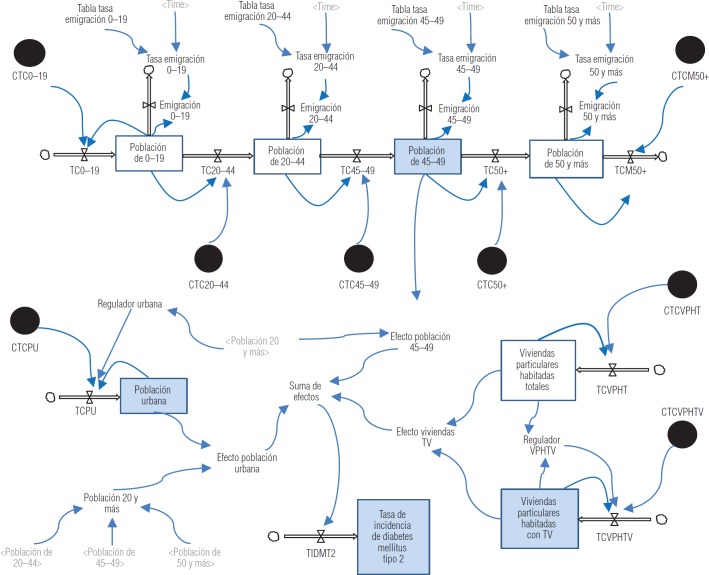
Modelo dinámico predictivo de la tasa de incidencia de la diabetes mellitus tipo 2

El modelo dinámico predictivo muestra los flujos de cada indicador (población de 45-49, población urbana y viviendas particulares habitadas que cuentan con televisión) hacia su respectivo efecto, el de estos hacia la suma de efectos y finalmente este flujo hacia la TIDM2 (figura 2).

**FIGURA 3.**
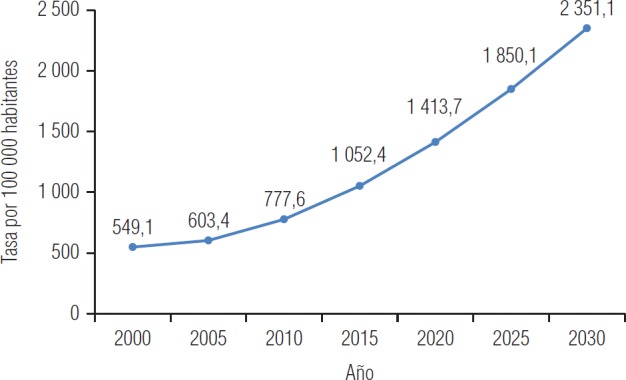
Escenario futuro probable de la tasa de incidencia de la diabetes mellitus tipo 2 en población ≥ 20 años de edad

**FIGURA 4.**
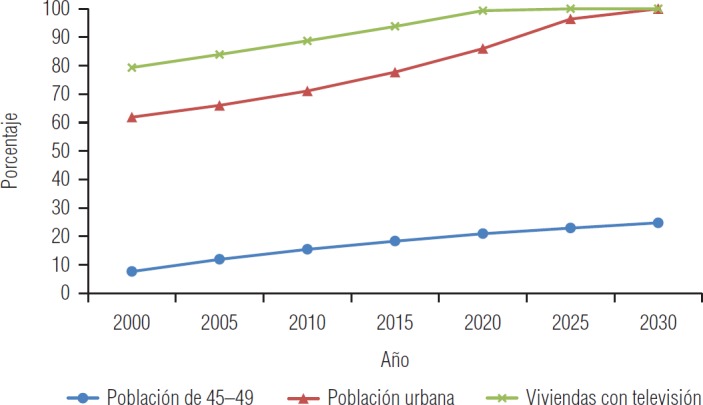
Escenarios futuros probables de los factores de riesgo de la diabetes mellitus tipo 2

En el escenario de la TIDM2, que es el punto medular de este análisis, se observa un crecimiento casi exponencial de 549,1 por 100 000 en 2000 a 2 351,1 en 2030 (figura 3). Por otra parte, los tres escenarios de los indicadores analizados también muestran un incremento sostenido casi exponencial (figura 4).

Los submodelos de los indicadores población de 45-49 años y de la población urbana tuvieron un buen ajuste, con coeficientes de correlación r = 0,80 y r = 0,82, respectivamente. Sin embargo, el ajuste de los indicadores viviendas particulares habitadas que cuentan con televisión y TIDMT2 fue regular. Las pendientes estimadas fueron las siguientes: TIDM2= 61,54 por 100 000 habitantes, población de 45-49 años = 0,55%, población urbana = 1,44%, y viviendas particulares habitadas que cuentan con televisión = 0,75%.

***Fuente:*** elaboración propia.

## DISCUSIÓN

Según los resultados de la regresión lineal múltiple, la TIDM2 depende significativamente, de forma exploratoria, del grupo de edad de 45-49 años, de las viviendas particulares habitadas que cuentan con televisión y de la población urbana. Este resultado es congruente con los de otros estudios, en los cuales estos tres indicadores se consideran factores de riesgo de la diabetes (6-9). Por otro lado, los estimadores en unidades no estándar del modelo estructural confirmatorio se interpretan así: por cada unidad porcentual que incrementan las viviendas particulares habitadas que cuentan con televisión, se estima que la TIDM2 aumenta 4,46 unidades, 2,84 por cada unidad porcentual que aumenta la población urbana, y 156,69 por cada unidad porcentual que lo hace la población de 45-49 años. Los tres indicadores en conjunto, considerando las sinergias entre ellos y el error de medición de la TIDM2, explican 27,2% de dicha tasa y el 72,8% restante se atribuye a otros factores. Aunque ya se han notificado estas tres variables como factores de riesgo de manera individual (6-9), en este estudio también se identificó su efecto conjunto como factores de riesgo. Este análisis es muy recomendable pues la presencia simultánea de varios factores de riesgo tiene un efecto no sólo aditivo, sino multiplicativo del riesgo de cada factor por separado (10).

Por otra parte, los factores de riesgo considerados en este estudio tienen una variación en el tiempo y el espacio; los resultados de los submodelos dinámicos individuales, por sí solos, muestran probables escenarios futuros que pueden apoyar la toma de decisiones.

El escenario del indicador población de 45-49 años señala un incremento claro, casi exponencial, de 7,6% en el año 2000 y un aumento esperado de 24,7% para 2030. Este último escenario es de alerta, ya que en este estudio resultó ser el factor de riesgo de más jerarquía; aunque este grupo de edad no se ha descrito como el más afectado en esta investigación, sí es el único grupo de edad en el cual se detectó una relación estadísticamente significativa con la TIDM2. Además, el escenario es congruente (r = 0,80) con las proyecciones oficiales del Consejo Nacional de Población de México (CONAPO) en un 80% (19).

Por otro lado, el porcentaje de viviendas particulares habitadas que cuentan con televisión tuvo un crecimiento sostenido, desde 79,3% en el año 2000 a 100% que se espera que alcance aproximadamente en 2025. Este indicador se identificó como el segundo factor de riesgo más importante: ver la televisión de manera prolongada provoca sedentarismo, otra variable que, a su vez, se identificó como factor de riesgo de la diabetes (9).

***Fuente:*** elaboración propia.

***Nota:*** las constantes de las tasas de cambio son: CTC0-19: de la población de 0 a 19 años de edad; CTC20-44: de la población de 20 a 44 años de edad; CTC45-49: de la población de 45 a 49 años de edad; CTC50+: de la población de 50 y más años; CTCM50+: de la mortalidad en población de 50 y más años; CTCPU: de la población urbana de 20 y más años; CTCVPHT: de las viviendas particulares habitadas totales; CTCVPHTV: de las viviendas particulares habitadas que cuentan con televisión; Tasas de cambio TC0-19: de la población de 0 a 19 años de edad; TC20-44: de la población de 20 a 44 años de edad; TC45-49: de la población de 45 a 49 años de edad; TC50+: de la población de 50 y más años; TCM50+: de la mortalidad en población de 50 y más años; TCPU: de la población urbana de 20 y más años; TCVPHT: de las viviendas particulares habitadas totales; TCVPHTV: de las viviendas particulares habitadas que cuentan con televisión, y TIDMT2: de la incidencia de diabetes mellitus tipo 2.

Asimismo, el escenario del indicador población urbana muestra un incremento claro, casi exponencial: de 27,1% en el año 2000, se espera un 58,0% para el año 2030. Este escenario requiere suma atención, pues en este estudio resultó ser el tercer factor de riesgo con más jerarquía. La población que vive en zonas urbanas ya se ha considerado factor de riesgo de la diabetes con anterioridad (7,8), toda vez que los estilos de vida en el medio urbano son menos saludables que en el rural (21).

En el escenario de la TIDM2 se observa un crecimiento exponencial (de 549,1 en el año 2000 a 2 351,1 en 2030). Esto es el resultado de la interacción de los tres factores analizados, considerando la variación de los mismos en el tiempo: el peso que cada uno de ellos representa en la TIDM2 y la interacción entre los tres explican 27,2% de dicha tasa. La tendencia mostrada por el escenario futuro coincide con lo publicado por la Encuesta Nacional de Salud y Nutrición en México (ENSANUT), donde se notificó un incremento de la prevalencia de 3,8% en la población de 20 y más años de edad en el Estado entre 2006 y 2012 (3).

Este resultado no indica que se tenga la certeza de que el escenario va a suceder, pero proporciona una aproximación de la realidad de este grave problema de salud, que es probable si los factores que determinan la diabetes mellitus mantienen la tendencia observada y estimada. En orden jerárquico descendente, los factores de riesgo que determinan la TIDM2 son, por consiguiente la población de 45-49 años, las viviendas particulares habitadas que cuentan con televisión y la población urbana.

***Fuente:*** elaboración propia.

***Fuente:*** elaboración propia.

Es fundamental que las autoridades de salud de San Luis Potosí y de México consideren la necesidad de reestructurar las políticas sanitarias con la finalidad de hacer eficiente su aplicación, generar acciones y estrategias que garanticen la prevención de la diabetes mellitus tales como la coordinación entre los distintos sectores (educativo, económico y social) y concienticen a las familias para que tengan estilos de vida saludables.

Otro aspecto de suma importancia es el factor de riesgo identificado en la población de 45 a 49 años de edad, ya que es importante resaltar que de seguir su tendencia afectará seriamente la economía no sólo del estado de San Luís Potosí, sino de México, debido a que es un grupo que se encuentra en edad productiva. Asimismo, el gasto que se generará por la atención de su salud rebasará las capacidades técnicas y financieras del estado. Por ello, se deben implementar acciones específicas dirigidas a esta población. Además, la vinculación del sector laboral gubernamental y no gubernamental es clave para desplegar estrategias claras y viables que incluyan las acciones dirigidas a mejorar los estilos de vida y transformarlos en saludables.

Las principales limitaciones de este estudio fueron los subregistros de la TIDM2 y la falta de datos oficiales de factores de riesgo como la obesidad, el sedentarismo y el consumo de calorías, entre otros, lo cual dificulta la integridad de los análisis. Otra limitante fue el acceso reducido y el escaso número de registros especialmente en las viviendas particulares que cuentan con televisión.

Como conclusión puede afirmarse que el escenario de la TIDM2 muestra un crecimiento exponencial del año 2000 al 2030, lo cual traduce la interacción de los tres factores de riesgo analizados y ordenados por jerarquía según el peso que tiene en el desarrollo de la enfermedad: el grupo de edad 45-49 años, las viviendas particulares habitadas que cuentan con televisión y la población urbana. El efecto conjunto de estos tres factores explica 27,2% de la TIDM2. El modelo dinámico aplicado es útil para realizar análisis multivariantes con diferentes niveles de profundidad, como el exploratorio y el confirmatorio, y para desarrollar modelos dinámicos holísticos predictivos. También puede ser útil aplicarlo en otras enfermedades.

Para futuros estudios se recomienda seleccionar otros indicadores que estén disponibles para todo el periodo que se desea analizar, como los antecedentes heredofamiliares, el patrón en el consumo diario de calorías o el régimen de ejercicio, entre otros. Sería útil asimismo ampliar los años del periodo de estudio, así como el tamaño de la muestra para fortalecer los análisis.

### Financiación.

Este estudio no recibió financiación.

### Declaración.

Las opiniones expresadas por los autores son de su exclusiva responsabilidad y no reflejan necesariamente los criterios ni la política de la Organización Panamericana de la Salud o de la *RPSP/PAJPH*.
